# Intact interferon signaling in peripheral blood leukocytes of high-grade osteosarcoma patients

**DOI:** 10.1007/s00262-012-1232-6

**Published:** 2012-03-10

**Authors:** Emilie P. Buddingh, S. Eriaty N. Ruslan, Dagmar Berghuis, Hans Gelderblom, Jakob K. Anninga, Pancras C. W. Hogendoorn, R. Maarten Egeler, Marco W. Schilham, Arjan C. Lankester

**Affiliations:** 1 Department of Pediatrics, J6-S, Leiden University Medical Center, PO Box 9600, 2300 RC Leiden, The Netherlands; 2 Department of Pathology, Leiden University Medical Center, Leiden, The Netherlands; 3 Department of Clinical Oncology, Leiden University Medical Center, Leiden, The Netherlands

**Keywords:** Osteosarcoma, Tumor immunology, Interferon-α, NK cell

## Abstract

High-grade osteosarcoma has a poor prognosis with an overall survival rate of about 60 percent. The recently closed European and American Osteosarcoma Study Group (EURAMOS)-1 trial investigates the efficacy of adjuvant chemotherapy with or without interferon-α. It is however unknown whether the interferon-signaling pathways in immune cells of osteosarcoma patients are functional. We studied the molecular and functional effects of interferon treatment on peripheral blood lymphocytes and monocytes of osteosarcoma patients, both in vivo and ex vivo. In contrast to other tumor types, in osteosarcoma, interferon signaling as determined by the phosphorylation of signal transducer and activator of transcription (STAT)1 at residue 701 was intact in immune cell subsets of 33 osteosarcoma patients as compared to 19 healthy controls. Also, cytolytic activity of interferon-α stimulated natural killer cells against allogeneic (*n* = 7 patients) and autologous target cells (*n* = 3 patients) was not impaired. Longitudinal monitoring of three osteosarcoma patients on interferon-α monotherapy revealed a relative increase in the CD16-positive subpopulation of monocytes during treatment. Since interferon signaling is intact in immune cells of osteosarcoma patients, there is a potential for indirect immunological effects of interferon-α treatment in osteosarcoma.

## Introduction

High-grade osteosarcoma is a primary malignant bone tumor with an overall survival rate of about sixty percent [1]. Intensification of chemotherapeutic treatment has not led to improved outcome, and other therapeutic modalities are currently under investigation [2]. The recently closed for accrual European and American Osteosarcoma Study Group (EURAMOS)-1 trial investigates whether maintenance treatment with interferon(IFN)-α-2b after standard chemotherapy for osteosarcoma patients who have a good response to preoperative chemotherapy (i.e., >90% necrotic tumor tissue) is effective [3, 4]. IFN-α has proven anti-tumor effect in several tumor types, such as hairy cell leukemia and renal cell cancer, and has been used in osteosarcoma patients as adjuvant treatment in Scandinavia since the 1970s [5–7].

The anti-proliferative effect of IFN-α on osteosarcoma cells has been shown in vitro and in a xenograft model in nude mice [8, 9]. Also, expression of IFN receptors on human osteosarcoma cells is associated with a better prognosis [10]. However, IFN-α can also exert indirect anti-tumor activity; for example, through immunostimulatory effects. The type I IFNs (IFN-α and -β) binds to the IFN type I receptor consisting of the two subunits IFNAR1 and IFNAR2, which is expressed by immune cells such as natural killer (NK) cells and monocytes [11, 12]. Indirect anti-tumor effects of type I IFNs were essential for the clearance of immunogenic sarcomas in IFNAR1 deficient mice, since it was dependent on the expression of IFNAR1 on hematopoietic host cells and not on tumor cells [13]. Whether similar indirect anti-tumor effects also occur in the treatment for human osteosarcoma with type I IFNs is unknown. The binding of type I and type II (IFN-γ) IFNs to their respective receptors results in the activation of Janus Kinase (JAK), subsequent phosphorylation of signal transducer and activator of transcription (STAT) and finally transcription of target genes. Phosphorylation of STAT1 at tyrosine residue 701 occurs rapidly following receptor–ligand interaction and is critical for both type I and type II IFN signaling [14]. IFN signaling as determined by STAT1 phosphorylation was impaired in lymphocytes of patients suffering from breast cancer, melanoma, and gastrointestinal cancer [15–18]. Impaired IFN signaling may be rescued at the level of JAK-1 induced STAT1 phosphorylation; for example, by interleukin (IL)-12 pre-treatment, as was shown in a murine melanoma model [19].

Here, we addressed the molecular and functional effects of IFN treatment on immune cell subsets of osteosarcoma patients, both in vivo and ex vivo. To interpret the future results of the IFN-α treatment arm of the EURAMOS-1 trial, it is essential to know whether IFN-signaling pathways in immune cells of osteosarcoma patients are intact.

## Patients and methods

### Patients

Peripheral blood mononuclear cells (PBMCs) of 33 newly diagnosed osteosarcoma patients and 19 healthy controls were available for flow cytometric evaluation of IFN-induced phosphorylation of STAT1 (Table 1). PBMCs of 7 patients and 7 controls were available for cytolytic experiments. From three osteosarcoma patients treated with IFN-α monotherapy following the completion of adjuvant chemotherapy, PBMCs were collected at diagnosis (prior to the start of chemotherapy), prior to the start of IFN-α monotherapy and at one or two time points during the first few weeks of treatment with IFN-α (subcutaneous PegIntron, 0.5 μg/kg/week for 4 weeks, then dose escalation to 1.0 μg/kg/week). PBMCs were obtained after written informed consent, approved by the Institutional Review Board. All samples were handled in a coded fashion.

**Table 1:** Clinicopathological details of osteosarcoma patients and healthy controls included

	Osteosarcoma	Healthy controls
*n*	33	19
Male	18 (54.5%)	12 (63.2%)
Age median (range)	16 (6–56)	17 (8–45)
*Location primary tumor*		
Distal femur	19 (57.6%)	
Prox tibia	6 (18.2%)	
Prox humerus	5 (15.2%)	
Other long bone	3 (9.1%)	
*Histological subtype*		
Conventional	29 (87.9%)	
Osteoblastic	24	
Chondroblastic	3	
Unusual	2	
Telangiectatic	4 (12.1%)	
High-grade surface	1 (3.0%)	

### Culture of cell lines and PBMCs

The cell line K562 (obtained from ATCC) and PBMCs were cultured in RPMI 1640 medium (Invitrogen, Carlsbad, CA) with 10% fetal calf serum (FCS, Invitrogen) and 1% penicillin/streptomycin (P/S, Invitrogen). PBMCs were isolated by Ficoll density gradient centrifugation and stored in liquid nitrogen. After thawing, cells were allowed to recover overnight, except when used for flow cytometric evaluation of dendritic cell (DC) activation, in which case, cells were analyzed immediately. For cytolytic assays, cells were cultured overnight with or without 100 IU/mL IFN-α (Roche, Basel, Switzerland). Prior dose-finding pilot experiments determined this dose to result in good cell viability and reproducible NK cell activation (data not shown). The primary cell culture L2635 was established from a pre-treatment biopsy of osteosarcoma patient 398 as described previously and maintained in RPMI 1640 with 20% FCS and 1% P/S [20].

### Flow cytometric evaluation of IFN-induced phosphorylation of STAT1

Cells were stained with surface staining antibodies in staining buffer (PBS with 0.05% bovine serum albumin without sodium azide) for 30 min at 37°C. Surface staining antibodies used for natural killer (NK) and T-cell subsets were fluorescein isothiocyanate (FITC)-conjugated anti-CD3 (349201, BD, San Diego, CA) and allophycocyanin (APC)-conjugated anti-CD56 (2474, IOtest/Immunotech, Marseille, France). Antibodies used for monocyte and B cell subsets were FITC-conjugated anti-CD20 and APC-conjugated anti-CD14 (345792 and 340436, BD). Cells were stimulated with or without 1,000 IU/mL IFN-α or IFN-γ for 15 min at 37°C and fixed in 4% paraformaldehyde, as previously described by Critchley-Thorne et al. [15]. Cells were permeabilized using ice-cold Perm Buffer III (BD Phosflow) for 30 min and stained for 60 min on ice with phycoerythrin (PE)-conjugated anti-STAT1 (directed against the N-terminus of STAT1 to determine total STAT1 levels, 558537, BD) and peridinin chlorophyll protein (PerCP)-Cy5.5-conjugated anti-pY701-STAT1 (directed against phosphorylated tyrosine residue 701 of STAT1 to determine the levels of phosphorylated STAT1, 560113, BD). Isotype control antibodies were used to correct for the background levels of fluorescence. All flow cytometric analyses were done on a FACScalibur with Cellquest software (both BD).

### Flow cytometric evaluation of PBMC subsets

PBMC subsets were determined as follows: T cells were CD3-PerCP-Cy5.5-positive (332771, BD), NK cells were CD3-PerCP-Cy5.5-negative, and CD56-PE-positive (R7251, Dako, Glostrup, Denmark), B-cells were CD20-FITC-positive (345792, BD), and monocytes were CD14-APC-positive (340436, BD). Myeloid dendritic cells (mDCs) were CD3-, CD19-, and CD14-negative (all PerCP-Cy5.5-conjugated, 332771, 332780, and 550787, BD) and BDCA-1- and BDCA-2-positive (PE-conjugated, Miltenyi Biotec, Bergisch Gladbach, Germany). Activation status of mDCs was determined by evaluating mean fluorescence intensities of HLA-DR-APC (347403, BD) and CD86-FITC (555657, BD). Plasmacytoid dendritic cells (pDCs) were CD3-, CD19-, and CD14-negative and BDCA-2-APC-positive (Miltenyi). Activation status of pDCs was determined with anti-CD86-FITC and anti-HLA-DR-PE (555657 and 347367, BD). Monocytes were evaluated by CD14-PerCP-Cy5.5, CD16-PE (347617, BD), HLA-DR-APC, and CD86-FITC.

### Cytolytic assays

Four hour chromium release cytolytic assays were performed as described earlier [20]. Briefly, target cells were incubated with 3.7 MBq sodium-51-chromate (PerkinElmer, Wellesley, MA) for 1 h. Effector cells (with or without overnight IFN-α stimulation) were incubated for 4 h with 2,500 target cells at eight effector:target (E:T) ratios in triplicate. Using these conditions, cytolysis observed is caused by NK cells, since antigen-specific T cells (which are present at low frequencies if at all) are insufficiently expanded. Therefore, the E:T ratios were corrected for the percentage of NK cells of PBMCs as determined by flow cytometry. Maximum and spontaneous release was determined by incubating targets in 2N HCl or medium, respectively. Supernatants were harvested and counted in a gamma counter (Wallac, PerkinElmer). Specific lysis was determined as: (experimental release-spontaneous release)/(maximum release-spontaneous release) × 100%.

### Statistical analysis

Statistical analyses were performed using GraphPad Prism 5.0 (LaJolla, CA). Two-sided *P* values lower than 0.05 were determined to be significant.

## Results and discussion

### Peripheral blood monocytes and lymphocytes of osteosarcoma patients have intact interferon-signaling ex vivo

**Fig. 1:**
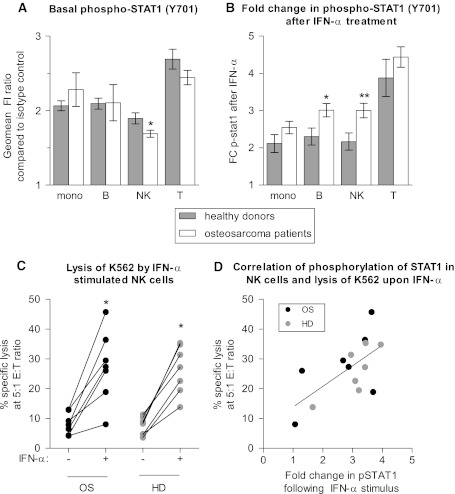
PBMCs of osteosarcoma patients are normally responsive to IFN-α. **a** Flow cytometric evaluation of basal levels of phosphorylated STAT1 in immune cell subsets shows slightly lower levels of phosphorylated STAT1 in NK cells of osteosarcoma patients at diagnosis (*n* = 33) as compared to healthy donors (*n* = 19). **b** Following an in vitro stimulus with 1,000 IU/mL IFN-α, hyperphosphorylation of B and NK cells of osteosarcoma patients was observed. For both **a** and **b**, *error bars* denote standard error of the mean; student’s *t*-test *P* value <0.05 noted as *; <0.01 = **. **c** Lysis of the allogeneic NK cell target K562 by PBMCs of healthy donors (HD) and osteosarcoma patients (OS) increased significantly following overnight culture in 100 IU/mL IFN-α (IFN-α +). Effector to target (E:T) ratio was corrected for percentage of NK cells. Lysis was similar by healthy donor and osteosarcoma patient derived cells. Wilcoxon signed ranked test; *P* value <0.05 noted as *. **d** Following overnight culture of PBMCs in IFN-α, percentage-specific lysis correlated with fold change in pSTAT1 in NK cells (*P* value 0.06, Spearman r 0.52)

Total STAT-1 levels were similar in peripheral blood subsets of osteosarcoma patients and healthy controls. To determine whether the IFN-signaling pathway was intact, we assessed STAT1 phosphorylation in IFN-stimulated immune cells of controls and patients. Basal levels of phospho-STAT1 were slightly lower in NK cells of osteosarcoma patients at diagnosis (*n* = 33) than in NK cells of healthy controls (*n* = 19; Fig. 1a), similar to what has been described for melanoma [21]. However, in contrast to what has been reported for melanoma, breast cancer, and gastrointestinal cancer, IFN signaling was intact in immune cells of osteosarcoma patients, as determined by a 15 min ex vivo stimulus with 1,000 IU/mL IFN-α or IFN-γ (results for IFN-α shown in Fig. 1, results for IFN-γ not shown). Responses were similar in almost all PBMC subsets for patients and controls, except for a hyperphosphorylation of STAT1 in B- and NK cells of osteosarcoma patients in response to IFN-α (Fig. 1b). The functional response of NK cells of osteosarcoma patients and healthy controls to IFN-α was similar, as both responded to overnight IFN-α culture with increased cytolytic capacity toward the NK cell target K562 (Fig. 1c). There was possibly some, but not statistically significant trend for correlation between phosphorylated STAT1 in NK cells and percentage of specific lysis of K562 (Fig. 1d, *P* value 0.06, Spearman r 0.52).

We previously showed higher cytolytic capacity of IL-15-activated NK cells of osteosarcoma patients than of healthy donors [20]. The binding of IL-15 and IFN-α to their respective receptors has the association of JAK1 with the receptors in common. Together with Tyk2, this results in phosphorylation of STAT1 in case of IFN-α-signaling and together with JAK3 in phosphorylation of STAT3 and STAT5 in case of IL-15 signaling. The hyperphosphorylation of STAT1 in response to IFN-α and the earlier reported increased cytolytic capacity in response to IL-15 in NK cells of osteosarcoma patients as compared to healthy donors could be due to increased functionality of the common upstream signaling molecule JAK1. Pro-inflammatory cytokines such as IL-12 potentiate JAK1 induced STAT phosphorylation [19]. However, levels of IL-12(p70) were similar in plasma of patients and controls, and IL-12(p70) levels did not correlate with IFN-α induced STAT1 phosphorylation (data not shown). Whether increased STAT1 phosphorylation in NK cells of osteosarcoma patients is related to differential expression or activity of JAK1 remains to be investigated.

### Phenotypic and functional analysis of PBMCs of osteosarcoma patients on IFN-α monotherapy

In three osteosarcoma patients who received IFN-*α* monotherapy following chemotherapeutic treatment according to the EURAMOS-1 protocol, detailed flow cytometric phenotypic analysis of PBMCs was performed during the first few weeks of IFN-α monotherapy. In all three patients, B cell numbers were very low (on average 3% of lymphocytes) at the end of chemotherapeutic treatment and during IFN treatment returned to levels similar to those at diagnosis, before start of chemotherapy (on average about 30% of lymphocytes or 500 cells/μL). No consistent changes were seen in percentage or absolute number of mDCs, pDCs, NK-, or T cells. The activation status of monocytes, mDCs, or pDCs did not change during the first weeks of treatment, as measured by the expression of CD86 and HLA class II.

**Fig. 2:**
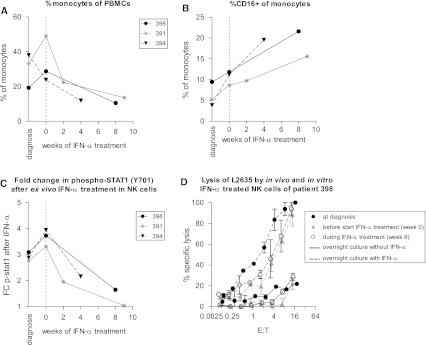
Effect of in vivo IFN-α administration on monocytes and NK cells. **a** Following in vivo treatment with IFN-α of three osteosarcoma patients, the percentage of monocytes of PBMCs decreased. **b** The relative percentage of CD16-positive monocytes increased. **c** NK cells which had been exposed to IFN-α in vivo showed less phosphorylation of STAT1 in response to ex vivo IFN-α stimulation. **d** Cytolysis of the autologous osteosarcoma target cell line L2635 by PBMCs of patient 398 did not change significantly during in vivo treatment with IFN-α (*solid lines*). Following overnight culture in 100 IU/mL IFN-α, lysis by PBMCs collected at all time points increased (*dashed lines*). Effector to target (E:T) ratio was corrected for the percentage of NK cells. *Error bars* denote standard error of the mean of experiment performed in triplicate

In all three patients, a reduction in percentage of monocytes was seen during the first 2–8 weeks of IFN treatment. However, the relative contribution of CD16+ monocyte subpopulation increased (Figs. 2a, b). CD16+ monocytes are considered “pro-inflammatory” because of the high expression of tumor necrosis factor (TNF)-α and low expression of IL-10 [22]. These pro-inflammatory monocytes may have anti-tumor effects, akin to what has been described for “pro-inflammatory” M1 type macrophages. We have recently demonstrated the prognostic significance of infiltrating macrophages in osteosarcoma [23]. Further studies are needed to determine whether IFN-induced phenotypic changes in peripheral blood monocytes are associated with changes in intratumoral macrophage number or phenotype, and whether this is associated with altered expression of specific cytokines. However, this will require IFNs to be administered earlier, before resection of the primary tumor or relapses.

During in vivo treatment with IFN-α, total STAT1 levels in monocytes, NK cells, and T cells increased, but the basal levels of phosphorylated STAT1 did not change in any of the leukocyte subsets (data not shown). NK- and T cells, which had been exposed to IFN-α in vivo, showed less induction of phosphorylation of STAT1 in response to ex vivo IFN-α stimulation (NK cells shown in Fig. 2c). Nevertheless, induction of cytolytic capacity against the NK cell target K562 and the osteosarcoma culture L2635 following overnight IFN-α stimulation of PBMCs was unimpaired, both against autologous and allogeneic targets (example shown for PBMCs of patient 398 and its autologous target cells L2635 in Fig. 2d), indicating that in vivo treatment with IFN-α has not rendered these cells functionally unresponsive to an additional ex vivo IFN stimulus.

The lack of induction of phosphorylated STAT1 during in vivo IFN-α treatment was surprising. There are several possible explanations. Phosphorylation of STAT1 is an event that occurs rapidly following IFN stimulus. Perhaps, STAT1 phosphorylation in vivo can be observed when PBMCs are analyzed within a few hours of first administration of IFN. Further studies are needed if this is indeed the case and if so, if this supposed the early activation of the JAK/STAT pathway in immune cells results in sustained activation of target genes and functional pathways. An alternative explanation might be that the level of IFN-induced activation of immune cells in vivo is dose-dependent and limited by toxic and adverse effects in vivo [24]. An additional in vitro stimulus was able to induce STAT1 phosphorylation and resulted in increased cytolytic NK cell activity, suggesting that in vivo IFN-α-induced immune cell activation might have been incomplete. On the other hand, it is still possible that the prolonged, relatively low level of IFN-α exposure in vivo on PBMCs does result in sustained anti-tumor immune effects; for example, through the observed changes in monocytes.

### Concluding remarks

Treatment for osteosarcoma patients with the anti-proliferative and immunomodulatory agent IFN-α could have direct anti-tumor and/or indirect immune-mediated effects. Here, we show IFN signaling to be intact in PBMCs of osteosarcoma patients, as determined by the phosphorylation of STAT1 and increased NK cell-mediated cytolysis in response to ex vivo IFN. Some of the experiments were performed on small sample numbers, but since results were consistent throughout, we believe the results to be valid in determining responsiveness of immune cells of osteosarcoma patients to IFN-α. Although the efficacy of IFN-α treatment in osteosarcoma will be assessed in the EURAMOS-1 trial, our data indicate that in contrast to some other tumor types, immune cells of osteosarcoma patients have retained IFN-responsiveness. Further clinical studies are needed to establish the extent and efficacy of indirect immunotherapeutic effects of IFN-α treatment in osteosarcoma patients.

## Abbreviations

APC: Allophycocyanin
E:T: Effector:target
EURAMOS: European and American Osteosarcoma Study Group
FCS: Fetal calf serum
FITC: Fluorescein isothiocyanate
HD: Healthy donor
IFN: Interferon
JAK: Janus Kinase
IL: Interleukin
mDC: Myeloid dendritic cell
NK: Natural killer
PBMC: Peripheral blood mononuclear cells
pDC: Plasmacytoid dendritic cell
PE: Phycoerythrin
PerCP: Peridinin chlorophyll protein
P/S: Penicillin/streptomycin
STAT: Signal transducer and activator of transcription
